# Lack of an Association between CYP11B2 C-344T Gene Polymorphism and Ischemic Stroke: A Meta-Analysis of 7,710 Subjects

**DOI:** 10.1371/journal.pone.0068842

**Published:** 2013-08-08

**Authors:** Yan Pi, Li-li Zhang, Kai Chang, Lu Guo, Yun Liu, Bing-hu Li, Xiao-jie Cao, Shao-qiong Liao, Chang-yue Gao, Jing-cheng Li

**Affiliations:** 1 Department of Neurology, Institute of Surgery Research, Daping Hospital, Third Military Medical University, Chongqing, China; 2 Department of Clinical Laboratory Medicine, Institute of Surgery Research, Daping Hospital, Third Military Medical University, Chongqing, China; The Scripps Research Institute, United States of America

## Abstract

**Background:**

The association between aldosterone synthase (CYP11B2) C-344T gene polymorphism and ischemic stroke remains controversial and ambiguous. To better explain the association between CYP11B2 polymorphism and ischemic stroke risk, a meta-analysis was performed.

**Methods:**

Based on comprehensive searches of Medline, Embase, Web of Science, CNKI and CBM databases, we identified and abstracted outcome data from all articles to evaluate the association between CYP11B2 polymorphism and ischemic stroke. The pooled odds ratios (ORs) with 95% confidence intervals (CIs) were performed in all genetic models. Fixed or random effects model was separately used depending on the heterogeneity between studies. Publication bias was tested by Begg's funnel plot and Egger's regression test.

**Results:**

A total of 12 studies including 3,620 ischemic stroke cases and 4,090 controls were identified. There was no statistical evidence of association between CYP11B2 C-344T polymorphism and ischemic stroke in all genetic models (allelic model: OR = 1.19, 95% CI = 0.95–1.49; additive model: OR = 1.43, 95% CI = 0.91–2.27; dominant model: OR = 1.30, 95% CI = 0.89–1.89; and recessive model: OR = 1.24, 95% CI = 0.96–1.60). On subgroup analysis by ethnicity, similarly results were found in both Asians and non-Asians. For Asians, the combined ORs and 95% CIs were (allelic model: OR = 1.07, 95% CI = 0.87–1.32; additive model: OR = 1.15, 95% CI = 0.77–1.71; dominant model: OR = 1.13, 95% CI = 0.92–1.38; and recessive model: OR = 1.09, 95% CI = 0.84–1.40). For none-Asians, the combined ORs and 95% CIs were (allelic model: OR = 1.58, 95% CI = 0.90–2.76; additive model: OR = 2.37, 95% CI = 0.79–7.05; dominant model: OR = 1.79, 95% CI = 0.77–4.19; and recessive model: OR = 1.80, 95% CI = 0.96–3.36).

**Conclusion:**

The present meta-analysis suggested that CYP11B2 C-344T polymorphism was unlikely contribute to ischemic stroke susceptibility.

## Introduction

The renin-angiotensin-aldosterone system (RAAS) is important for cerebrovascular research, because of its influence on blood pressure, vasoconstriction, thrombosis, and vessel wall damage [1]. Aldosterone, one of the main effectors of the RAAS, affects sodium balance, intravascular volume, and blood pressure [2], [3]. Aldosterone synthase (CYP11B2) is a key enzyme in the biosynthesis of aldosterone, the CYP11B2 gene located in 8q22 spans approximately 7 kb and contains 9 exons and 8 introns [4], [5]. CYP11B2, which belongs to the cytochrome P450 gene superfamily, is responsible for the last biochemical reaction step in vivo to synthesize aldosterone [6], [7]. The C-344T (rs1799998) variant is a common polymorphism in the promoter region of CYP11B2, which involves a C to T substitution in the steroidogenic transcription factor 1 binding site [8]. The C-344T polymorphism has been reported to associate with higher aldosterone synthase activity and enhanced aldosterone production [7], which in turn induces sodium and water retention, resulting in increased peripheral vascular resistance, and precipitation of adverse vascular remodeling, vasoconstriction, thrombosis, and vessel wall damage, and ultimately affects the odds of stroke development [9], [10], [11]. As for ischemic stroke, a variety of epidemiological studies have evaluated the role of CYP11B2 C-344T polymorphism, however, there were apparent discrepancies among the results of these association studies [1], [2],[6],[9],[12],[13],[14],[15],[16],[17],[18]. It is likely that CYP11B2 C-344T polymorphism may influence the susceptibility of ischemic stroke. The present meta-analysis was therefore designed to derive a more precise estimation of the association between CYP11B2 C-344T polymorphism and ischemic stroke.

## Materials and Methods

### Data sources and search strategy

This meta-analysis followed the Preferred Reporting Items for Systematic Reviews and Meta-analysis (PRISMA) criteria [19]. To identify potentially relevant articles and abstracts, two investigators (Y.P. and L.Z.) independently performed a systematic electronic literature from Medline, Embase, Web of Science, CNKI (National Knowledge Infrastructure) and CBM (Chinese BioMedical Literature Database) databases for original articles published before February 2013. The initial search used the MeSH terms “aldosterone synthase OR CYP11B2 OR rs1799998” AND “polymorphism OR variant OR mutation” AND “ischemic stroke OR cerebral infarction OR brain infarction OR stroke”. We reviewed the bibliographies of all selection articles to identify additional relevant studies.

### Selection of publications

Two reviewers (Y.P. and K.C.) independently screened titles and abstracts of all studies for relevancy. Disagreements were resolved by a third opinion (J.L.). The inclusion criteria were: (1) case-control studies to evaluate the association between CYP11B2 C-344T polymorphism and risk of ischemic stroke, (2) useful data including genotype number or frequency given, (3) studies clearly describe ischemic stroke diagnoses and the sources of cases and controls, (4) studies written in English and Chinese with full-text, (5) genotype distribution of controls in Hardy-Weinberg equilibrium (HWE). The exclusion criteria were: (1) not case-control studies, (2) studies without available genotype number or frequency, (3) genotype distribution of controls not in HWE, (4) animal studies, reviews, case reports, and abstracts. For the studies with the same or overlapping data by the same authors, the most recent or largest population was selected.

### Data Extraction

Data were extracted independently from each study based on the inclusion and exclusion criteria listed above. Agreement was reached after discussion for conflicting data. The following data were collected from each study: first author, publication year, original country, ethnicity, sample size, mean age, genotyping method, and genotype number in cases and controls. Different ethnicities were categorized as Asian, and non-Asian. Study design was stratified to population-based (PB) studies and hospital-based (HB) studies.

### Quality Assessment

The quality of included studies was assessed independently using the Newcastle-Ottawa Scale (NOS) [20]. The NOS uses a ‘star’ rating system to judge quality based on 3 aspects of the study: selection, comparability, and exposure. Scores were ranged from 0 stars (worst) to 9 stars (best). Studies with a score ≥7 were considered to be of high quality. Disagreement was settled as described above.

### Statistical Analysis

The strength of association between CYP11B2 C-344T polymorphism and ischemic stroke risk was estimated by odds ratios (ORs) and corresponding 95% confidence intervals (CIs). The pooled ORs were calculated respectively for allelic model (T vs. C), additive model (TT vs. CC), dominant model (TT+TC vs. CC), and recessive model (TT vs. TC+CC). Heterogeneity assumption was assessed by the Q test and I^2^ statistics. If the result of the Q test was P_Q_ >0.1 and I^2^ <50%, the fixed-effects model was used to calculate the pooled ORs [21]; otherwise, the random-effects model was adopted [22]. Subgroup analysis was performed by ethnicity of study population, and meta-regression was used to analyze the sources of heterogeneity.

HWE was assessed using the Fisher's exact test, with the significance level was set at P<0.05. Sensitivity analysis was performed based on the high quality studies (according to the NOS score ≥7) and control source. Publication bias was tested by Begg's funnel plot and Egger's linear regression test (P<0.05 was considered representative of statistically significant publication bias) [23]. Data were analyzed by using STATA 11.0 (Stata Corporation, College Station, TX, USA) and Revman 5.0 (The Cochrane Collaboration).

## Results

### Study characteristics

The initial search identified one hundred and one potentially relevant publications, eleven of which met the inclusion criteria in the final. A flowchart detailing the process for study identification and selection was shown in Figure S1. The publication of Tu et al. [14] presented two separate case-control studies, each study in one publication was considered separately for pooling analysis. Therefore, eleven publications including 12 studies were involved in this meta-analysis, involving 3,620 ischemic stroke cases and 4,090 controls [1], [2], [6], [9], [12], [13], [14], [15], [16], [17], [18]. The main characteristics of the studies were summarized in Table 1. The sample sizes ranged from 123 to 1259 individuals (median 645.5, IQR 296.5–887.75), and the proportion of male patients ranged from 53.3% to 73.9% (median 64.35%, IQR 60.5%–70.3%). The NOS scores ranged from 6 to 9 (median 7.5, IQR 7–9), indicating that the methodological quality was generally good. The quality assessment of each included studies was shown in Table S1. Among the 12 studies, nine focused on Asians, and three on none-Asians.

**Table 1:** Main characteristics of studies included in the meta-analysis.

First author	Year	Region	Ethnicity	Male patients %	Mean age (years)		Control source	Genotype distribution cases/controls, N			Score	HWEY/N (P)
					Cases	Controls		CC	TC	TT		
Yan GH	2012	China	Asian	60.2	66.7±10.5	65.5 ±5.9	PB	14/18	118/118	200/114	9	Y(0.09)
Kim SK	2012	Korea	Asian	53.3	64.6±13.1	59.1±11.9	PB	5/31	46/143	68/146	8	Y(0.64)
Wu XM	2012	China	Asian	65.4	64.3±11.9	65.1±8.5	PB	10/6	38/62	59/74	8	Y(0.11)
Tu YC (a)	2011	China	Asian	64.7	61.1±9.50	62.2±9.3	NG	42/57	200/232	316/268	7	Y(0.52)
Tu YC (b)	2011	China	Asian	67.6	63.4±10.2	61.3±8.7	NG	46/68	225/270	294/356	6	Y(0.11)
Saidi S	2010	Tunisia	African	54.7	61.8 ±12.2	60.3 ±12.8	PB	68/212	166/181	95/51	9	Y(0.20)
Munshi A	2010	India	Caucasian	71.2	49.3 ±17.3	47.0±16.8	PB	51/87	179/195	173/112	9	Y(0.90)
Huriletemuer H	2010	China	Asian	64.0	60.0±10.0	53.0±11.0	HB	3/9	33/42	32/45	7	Y(0.86)
Zhao L	2010	China	Asian	71.4	64.0±10.9	64.7±13.2	HB	3/6	18/32	21/43	7	Y(0.99)
Wu XY	2008	China	Asian	62.3	67.3±13.5	63.9±10.9	HB	28/23	130/133	139/168	7	Y(0.63)
Wang XY	2006	China	Asian	73.9	64.8±11.2	63.5±10.2	PB	34/22	130/92	177/215	9	Y(0.19)
Brenner D	2005	France	Caucasian	61.4	69 (20–86)	68 (20–89)	HB	92/80	223/236	144/143	7	Y(0.30)

Abbreviations: PB, population-based; HB, hospital-based; NG, not given; HWE: Hardy-Weinberg equilibrium, Y: yes, N: no.

### Quantitative synthesis

**Figure 1:**
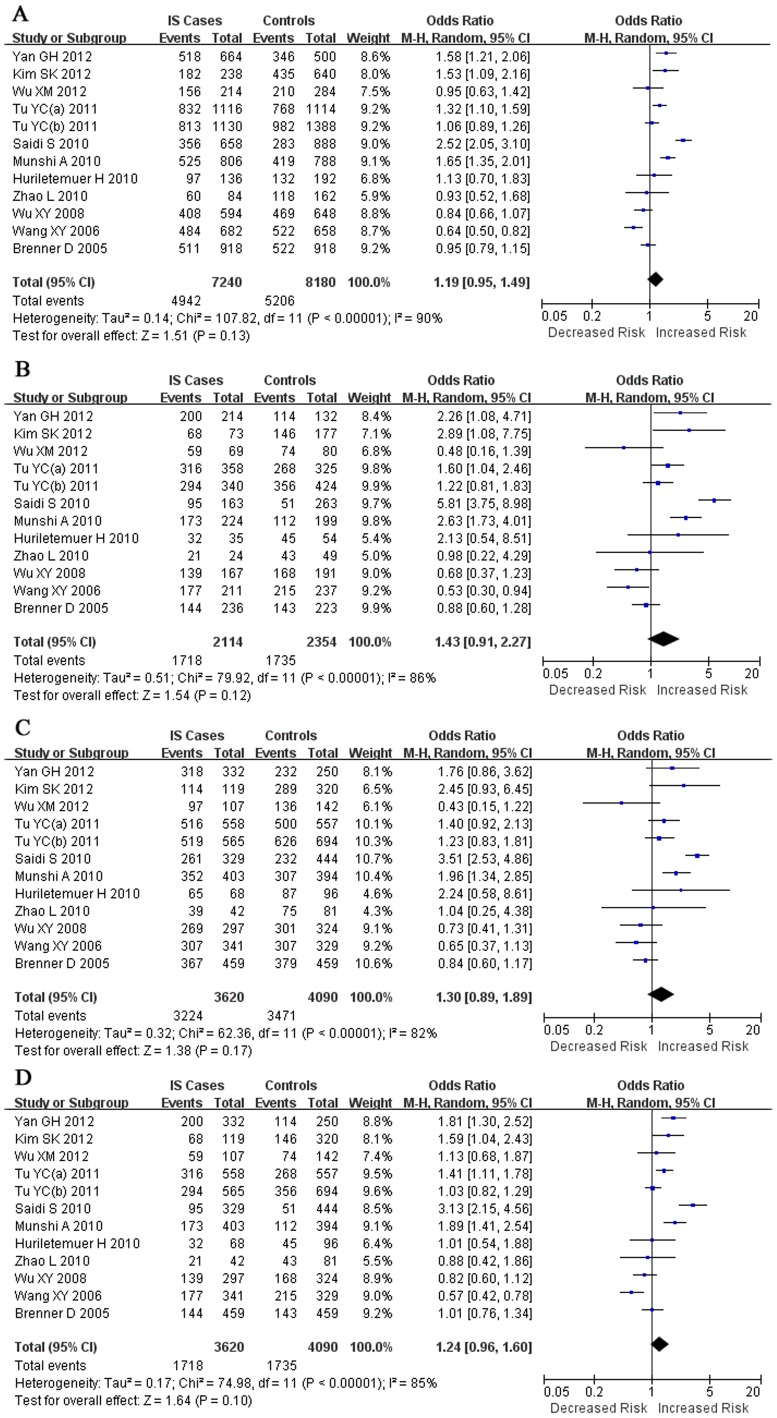
Forest plots for overall studies. No significant association was found between the CYP11B2 C-344T polymorphism and ischemic stroke in all genetic models. A: allelic model (T vs. C), B: additive model (TT vs. CC), C: dominant model (TT+TC vs. CC), D: recessive model (TT vs. TC+CC).

Random effects models were used to calculate the pooled ORs in all genetic models. Overall, the combined results showed no significant association between the CYP11B2 C-344T polymorphism and ischemic stroke for all genetic models (allelic model: OR = 1.19, 95% CI = 0.95–1.49; additive model: OR = 1.43, 95% CI = 0.91–2.27; dominant model: OR = 1.30, 95% CI = 0.89–1.89; and recessive model: OR = 1.24, 95% CI = 0.96–1.60). (Figure 1)

On subgroup analysis by ethnicity of study population, no evidence of association was also found in all genetic models. For Asians, the combined ORs and 95% CIs were (allelic model: OR = 1.07, 95% CI = 0.87–1.32; additive model: OR = 1.15, 95% CI = 0.77–1.71; dominant model: OR = 1.13, 95% CI = 0.92–1.38; and recessive model: OR = 1.09, 95% CI = 0.84–1.40). For none-Asians, the combined ORs and 95% CIs were (allelic model: OR = 1.58, 95% CI = 0.90–2.76; additive model: OR = 2.37, 95% CI = 0.79–7.05; dominant model: OR = 1.79, 95% CI = 0.77–4.19; and recessive model: OR = 1.80, 95% CI = 0.96–3.36). (Table 2)

**Table 2:** Subgroup analysis of CYP11B2 C-344T polymorphism and ischemic stroke in all genetic models.

Category	Allelic model	Additive model	Dominant model	Recessive model
	OR (95%CI) I^2^ (%)	OR (95%CI) I^2^ (%)	OR (95%CI) I^2^ (%)	OR (95%CI) I^2^ (%)
Ethnicity				
Asians	1.07 (0.97–1.32) 79	1.15 (0.77–1.71) 64	1.13 (0.92–1.38) 48	1.09 (0.84–1.40) 78
none-Asians	1.58 (0.90–2.76) 96	2.37 (0.79–7.05) 95	1.79 (0.77–4.19) 95	1.80 (0.96–3.36) 92

Abbreviations: OR, odds ratio; 95% confidence interval, 95% CI.

### Heterogeneity Analysis

Significant heterogeneity was found under the allelic (I^2 = ^90%, P_Q_ = 0.13), additive (I^2 = ^86%, P_Q_ = 0.12), dominant (I^2 = ^82%, P_Q_ = 0.17), and recessive (I^2 = ^85%, P_Q_ = 0.10) genetic models. To explore the sources of heterogeneity, the following meta-regression (including the control source, NOS scores, total sample size, and the TT, TC, and CC genotype number of ischemic stroke and control group sample size) was subsequently conducted in all genetic models. Under the allelic model, the heterogeneity could be explained by TT genotype number of ischemic stroke group sample size (TT1, P = 0.033), and TT genotype number of control group sample size (TT0, P = 0.002). Under the additive model, the heterogeneity could be explained by TT1 (P = 0.027) and TT0 (P = 0.005). Under the dominant genetic model, the heterogeneity could be explained by TT1 (P = 0.033) and TT0 (P = 0.011). Under the recessive model, the heterogeneity could be explained by TT0 (P = 0.005). (Table 3)

**Table 3:** Meta-regression results for the association of CYP11B2 C-344T gene under all genetic models.

Items	Coefficient	Standard Error	T value	P value	95% CI
allelic model					
TT1	4.678345	1.468548	3.19	0.033*	0. 6010015∼8.755689
TT0	−4.567678	0.6450131	−7.08	0.002*	−6.358522∼−2.776835
_cons	0.0487024	2.495109	0.02	0.985	−6.87883∼6.976235
additive model					
TT1	12.26766	3.613993	3.39	0.027*	2.233607∼22.30172
TT0	−7.861141	1.426674	−5.51	0.005*	−11.82222∼−3.900058
_cons	−6.384185	6.156214	−1.04	0.358	−23.47658∼10.70821
dominant model					
TT1	10.9838	3.422703	3.21	0.033*	1.480854∼20.48675
TT0	−5.706569	1.279871	−4.46	0.011*	−9.26006∼−2.153078
_cons	−7.427344	5.811689	−1.28	0.270	−23.56318∼8.708491
recessive model					
TT0	−5.447333	0.959107	−5.68	0.005*	−8.110242∼−2.784424
_cons	1.871771	3.360138	0.56	0.607	−7.457467∼11.20101

* P<0.05.

Coefficient: regression coefficient.

Abbreviations: Coefficient, regression coefficient; TT1, TT genotype number of ischemic stroke group sample size; TT0, TT genotype number of control group sample size; cons, constant item.

### Sensitivity Analysis

Robustness of our results with regard to different assumptions was examined by performing a sensitivity analysis. We carried out sensitivity analysis for CYP11B2 C-344T polymorphism by limiting the meta-analysis to the high NOS score (≥7) and basing on control source (PB studies). One study with relatively low NOS score (<7) and six studies without PB study design were excluded from the sensitivity analysis. The sensitivity analysis indicated that our data were stability and liability in this meta-analysis. The results were shown in Table S2.

### Publication bias

**Figure 2:**
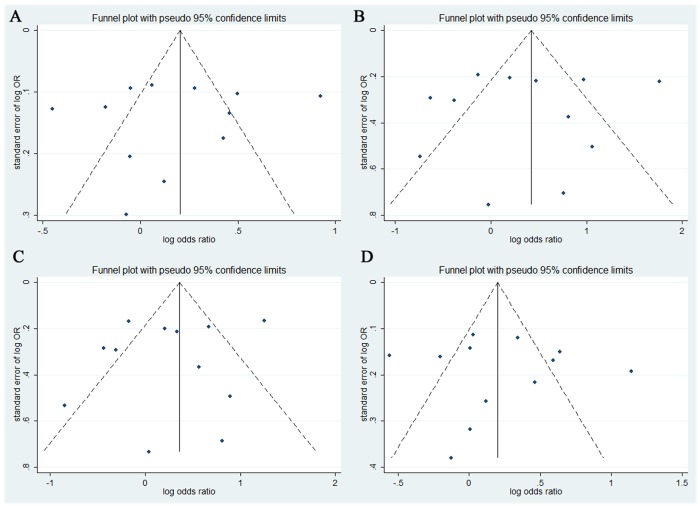
Funnel plots for overall studies. The shapes of the funnel plots did not reveal any evidence of obvious asymmetry.

The shapes of the funnel plots did not reveal any evidence of obvious asymmetry (Figure 2). Also there was no statistical evidence of publication bias among studies by using Egger's linear regression test (allelic model, P = 0.721; additive model, P = 0.721; dominant model, P = 0.486; and recessive model, P = 0.842).

## Discussion

Stroke is a multifactorial and polygenic disease with major clinical manifestations and multiple aetiologies, and a significant cause of disability and death in developed countries [24], [25]. The host genetic susceptibility, combined with environmental and clinical risk factors, may play a crucial role in the development and progression of ischemic stroke [26], [27], [28]. Recently, a variety of studies have focused on the association between CYP11B2 C-344T polymorphism and ischemic stroke. However, inconclusive results have been obtained and a single study may be too underpowered to detect a possible small effect of the gene polymorphism on ischemic stroke, especially when the sample size is relatively small. To better explain the association between CYP11B2 C-344T polymorphism and ischemic stroke risk, the present meta-analysis was then performed.

In the current meta-analysis, a total of twelve studies involving 7,710 subjects were included. No statistically significant evidence of an association between CYP11B2 C-344T polymorphism and ischemic stroke was found in the overall study population. The limited statistical findings may be arguably due to the differences of the ethnic population included in the meta-analysis, because different racial or ethnic populations have different frequencies of alleles, and different genetic backgrounds may affect ischemic stroke susceptibilities. So, subgroup analysis was performed based on ethnicity, and the results also demonstrated no significant differences.

Considering the heterogeneity under all genetic models, a meta-regression was conducted to explore the sources of heterogeneity. In the heterogeneity analysis, TT1 and TT0 were found to be the possible sources of heterogeneity under the allelic, additive, and dominant genetic models, and TT0 was found to be the possible sources of heterogeneity under the recessive genetic model. Sensitivity analysis was performed based on the high NOS score and control source, and no statistically significant evidence of an association between CYP11B2 C-344T polymorphism and ischemic stroke was also found. The results of sensitivity analysis indicated the present meta-analysis was stable and reliable.

In view of the complex effect of genetic polymorphisms on ischemic stroke progression, the lack of an association between CYP11B2 polymorphism and ischemic stroke susceptibility may attribute to other polymorphisms in RAAS (angiotensinogen T174M and M235T, angiotensin-converting enzyme I/D and 4656 2/3CT repeat, angiotensin II type 1 receptors A1166C and A153G, chymase G1903A), which could affect the expression of aldosterone and cerebrovascular disease susceptibility [1], [29], [30]. Brenner et al [1]. found that the polymorphisms of angiotensin II type 1 receptors A1166C and angiotensinogen T174M possibly influence the risk of ischemic stroke. Meanwhile, it has been reported that the interaction of CYP11B2, angiotensin II type 1 receptors, and angiotensinogen could influence the development of renal insufficiency in essential hypertension [31]. The interactions of the CYP11B2 C-344T polymorphism, age, and smoking status were also associated with enhanced risk of coronary artery disease [32]. Thus, the interactions among gene-gene and gene-environment might play a crucial role in the association between CYP11B2 polymorphism and ischemic stroke susceptibility.

For better interpreting the results, some limitations of our meta-analysis should be noted. Firstly, the retrieved literature is potentially not comprehensive enough. We did not track the unpublished articles to obtain data for analysis. The potential effect of this publication bias is unknown. Secondly, the small number of studies and sample sizes limited the ability to draw more solid conclusions. So, a larger number of available studies with a large sample size and adequate representation of ethnicities will help us to determine a more confident estimate of effect of this polymorphism on ischemic stroke. Thirdly, ischemic stroke is a multifactorial disease, and potential interactions between gene-gene and gene-environment should be considered.

In conclusion, the present meta-analysis provides evidence that CYP11B2 C-344T polymorphism was unlikely to be associated with genetic susceptibility of ischemic stroke based on the current published studies. Further studies with large sample size of different ethnic populations are required.

## Supporting Information

Figure S1 **Flow diagram of the selection of eligible studies.**
(TIF)

Table S1 **quality assessment of included studies.**
(DOC)

Table S2 **Sensitivity analysis for CYP11B2 C-344T polymorphism and ischemic stroke.**
(DOC)

Checklist S1 **PRISMA 2009 Checklist.**
(DOC)
