# Supplementary figures and images for: Lack of an Association between CYP11B2 C-344T Gene Polymorphism and Ischemic Stroke: A Meta-Analysis of 7,710 Subjects
Source: PLoS One. 2013 Aug 8;8(8):e68842. doi: 10.1371/journal.pone.0068842 (PMC3738569; doi:10.1371/journal.pone.0068842)

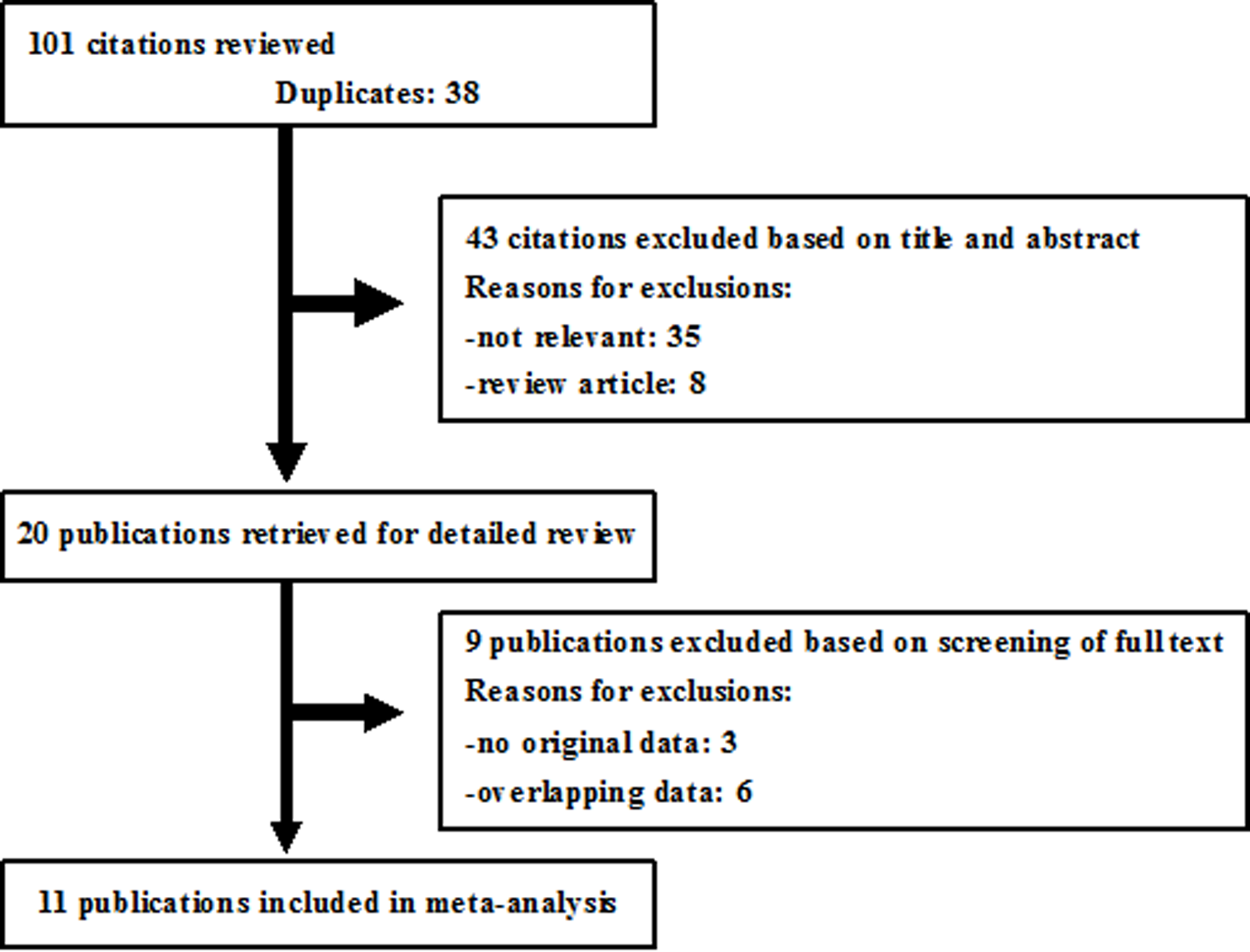

Supplement: Figure S1 — Flow diagram of the selection of eligible studies. (TIF) [file pone.0068842.s001.tif]
