# Supplementary material for: Lack of an Association between CYP11B2 C-344T Gene Polymorphism and Ischemic Stroke: A Meta-Analysis of 7,710 Subjects
Source: PLoS One. 2013 Aug 8;8(8):e68842. doi: 10.1371/journal.pone.0068842 (PMC3738569; doi:10.1371/journal.pone.0068842)
Supplement: Table S1 — quality assessment of included studies. (DOC) [file pone.0068842.s002.doc]

**Table S1:** **quality assessment of included studies.**

| First author | Quality assessment for studies of case-control studies | | | | | | | | | |
| --- | --- | --- | --- | --- | --- | --- | --- | --- | --- | --- |
|  | **Selection** | | | |  | **Comparability** |  | **Exposure** | | |
|  | 1 | 2 | 3 | 4 |  | 5 |  | 6 | 7 | 8 |
| Yan GH | ☆ | ☆ | ☆ | ☆ |  | ☆☆ |  | ☆ | ☆ | ☆ |
| Kim SK | ☆ | ☆ | ☆ | ☆ |  | ☆ |  | ☆ | ☆ | ☆ |
| Wu XM | ☆ | ☆ | ☆ | ☆ |  | ☆ |  | ☆ | ☆ | ☆ |
| Tu YC (a) | ☆ | ☆ |  | ☆ |  | ☆ |  | ☆ | ☆ | ☆ |
| Tu YC (b) | ☆ | ☆ |  |  |  | ☆ |  | ☆ | ☆ | ☆ |
| Saidi S | ☆ | ☆ | ☆ | ☆ |  | ☆☆ |  | ☆ | ☆ | ☆ |
| Munshi A | ☆ | ☆ | ☆ | ☆ |  | ☆☆ |  | ☆ | ☆ | ☆ |
| Huriletemuer H | ☆ | ☆ |  | ☆ |  | ☆ |  | ☆ | ☆ | ☆ |
| Zhao L | ☆ | ☆ |  | ☆ |  | ☆ |  | ☆ | ☆ | ☆ |
| Wu XY | ☆ | ☆ |  | ☆ |  | ☆ |  | ☆ | ☆ | ☆ |
| Wang XY | ☆ | ☆ | ☆ | ☆ |  | ☆☆ |  | ☆ | ☆ | ☆ |
| Brenner D | ☆ | ☆ |  | ☆ |  | ☆ |  | ☆ | ☆ | ☆ |

**Note:** A study can be awarded a maximum of one star for each numbered within the Selection and Exposure categories. A maximum of two stars can be given for Comparability.

**Selection**

1. Is the case definition adequate?
   1. Yes, with independent validation ☆
   2. Yes, eg record linkage or based on self reports
   3. No description
2. Representativeness of the cases
   1. Consecutive or obviously representative series of cases ☆
   2. Potential for selection biases or not stated
3. Selection of controls
   1. Community controls ☆
   2. Hospital controls
   3. No description
4. Definition of controls
   1. No history of disease ☆
   2. No description of source

**Comparability**

1. Comparability of cases and controls on the basis of the design or analysis
   1. Study controls for (Selection the most important factor)☆
   2. Study controls for any additional factor ☆(This criteria could be modified to indicate specific control for a second important factor)

**Exposure**

1. Ascertainment of exposure
   1. Secure record (eg surgical records)☆
   2. Structured interview where blind to case/control status☆
   3. Interview not blinded to case/control status
   4. Written self report or medical record only
   5. No description
2. Same method of ascertainment for cases and controls
   1. Yes ☆
   2. No
3. Non-response rate
   1. Same rate for both groups ☆
   2. Non-respondents described
   3. Rate different and no designation
