# Supplementary material for: Lack of an Association between CYP11B2 C-344T Gene Polymorphism and Ischemic Stroke: A Meta-Analysis of 7,710 Subjects
Source: PLoS One. 2013 Aug 8;8(8):e68842. doi: 10.1371/journal.pone.0068842 (PMC3738569; doi:10.1371/journal.pone.0068842)
Supplement: Table S2 — Sensitivity analysis for CYP11B2 C-344T polymorphism and ischemic stroke. (DOC) [file pone.0068842.s003.doc]

**Table S2: Sensitivity analysis for CYP11B2 C-344T polymorphism and ischemic stroke.**

| Sensitivity analysis | Allelic model |  | Additive model |  | Dominant model |  | Recessive model |
| --- | --- | --- | --- | --- | --- | --- | --- |
|  | OR (95%CI) |  | OR (95%CI) |  | OR (95%CI) |  | OR (95%CI) |
| Based on score | 1.20 (0.93-1.55) |  | 1.45 (0.87-2.44) |  | 1.31 (0.86-2.00) |  | 1.26 (0.94-1.69) |
| Based control source | 1.36 (0.90-2.05) |  | 1.78 (0.79-4.00) |  | 1.50 (0.81-2.77) |  | 1.49 (0.91-2.45) |

Abbreviations: OR, odds ratio; 95% confidence interval, 95% CI.

Based on score : studies with score<7 were excluded. Based control source: studies without population based were excluded.
